# Drivers of geophagy by red brocket deer (*Mazama americana*) at Amazonian interior forest mineral licks

**DOI:** 10.1002/ece3.10968

**Published:** 2024-02-09

**Authors:** Brian M. Griffiths, Lesa G. Griffiths, Yan Jin, Michael P. Gilmore

**Affiliations:** ^1^ The Earth Commons – Georgetown University’s Institute for Environment & Sustainability Georgetown University Washington DC USA; ^2^ Department of Animal and Food Sciences University of Delaware Newark Delaware USA; ^3^ Department of Plant and Soil Sciences University of Delaware Newark Delaware USA; ^4^ School of Integrative Studies George Mason University Fairfax Virginia USA

**Keywords:** Amazon, cervid, conservation, ecology, geophagy, mammal, mineral, ruminant, salt lick

## Abstract

Mineral licks are key ecological components of the Amazon rainforest, providing critical dietary functions for herbivorous and frugivorous mammals and birds, which help maintain the structure and function of the forest itself through seed and nutrient dispersal. One of the most frequent visitors of interior forest mineral licks in the Amazon is the red brocket deer (*Mazama americana*), a large‐bodied ruminant frugivore and seed predator. While several hypotheses for the drivers of geophagy exist, including mineral supplementation, toxin adsorption, and habitat selection, robust data on geophagy for the red brocket deer for large numbers of mineral licks is nonexistent. We used soil data from 83 mineral licks in conjunction with camera trap data from 52 of those mineral licks and a mixed‐effects modeling approach to test the three proposed hypotheses of geophagy for the red brocket deer. We found that consumed soils at mineral licks had elevated concentrations of almost all major and minor biologically active minerals measured, including Ca, Na, Mg, K, Cu, Zn, and Mn. Model results suggest that all three hypotheses hold true to some extent for the red brocket deer, with the greatest support for the mineral supplementation hypothesis, in particular with respect to Mg, Ca, Na, Cu, and Zn. This study provides critical information on the feeding ecology of the red brocket deer in the wild, and the first robust analysis of geophagy of an Amazonian mammal involving a large sample size of interior forest mineral licks.

## Graphical abstract

Mineral licks are critical resources for herbivores across the world, including the Peruvian Amazon, but the services they provide visiting animals are largely unknown. We tested three hypotheses for geophagical behavior in the red brocket deer, one of the most common visitors to Amazonian mineral licks, at mineral licks in the Peruvian Amazon using camera traps, soil sampling, and a modeling framework. We found that all three hypotheses had at least some support, but the mineral supplementation hypothesis was the most heavily supported.

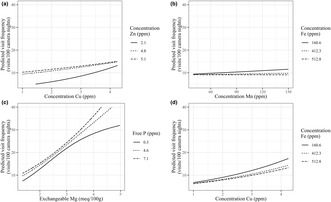

## 1 INTRODUCTION

The Amazon rainforest is one of the world's epicenters of biodiversity (Brown, 2014), and the conservation of that biodiversity has become a global priority (Schipper et al., 2008). Herbivorous and frugivorous mammals help maintain the structure and function of the Amazon rainforest by performing critical ecosystem services such as seed dispersal (Bodmer, 1991b; Brodie et al., 2009; Galetti et al., 2001) and nutrient transport (Doughty et al., 2016). These mammals also provide a source of food security for many rural populations that depend on subsistence hunting for animal protein (Alvard et al., 1995; Bizri et al., 2020; Griffiths, Bowler, et al., 2022; Mayor et al., 2021).

Soils in Amazonia are notoriously deficient in biologically active minerals, and many key herbivores and frugivores may be deficient in critical micro and macro nutrients needed for effective maintenance, reproduction, and growth. These species may compensate for mineral deficiencies in their diets by ingesting soil at natural mineral lick sites in the forest, a behavior known as geophagy (Abrahams & Parsons, 1996; Blake et al., 2011; Ferrari et al., 2008; Krishnamani & Mahaney, 2000). Alternative hypotheses for geophagy include toxin adsorption, where clays in ingested soils are assumed to bind toxic alkaloids from consumed plants and relieve gastrointestinal problems (Brightsmith et al., 2008; De Souza et al., 2002; Gilardi et al., 1999). A third plausible hypothesis which has received considerably less attention, and support, is the habitat selection hypothesis where animals may choose mineral licks based on their location, the suitability of the habitat they occur in, or the presence of predators in the habitat (Ayotte et al., 2008; Campbell et al., 2005; Tobler et al., 2009).

One of the most common visitors to interior forest Amazonian mineral licks is the red brocket deer (*Mazama americana*; Blake et al., 2011, 2013; Griffiths, Bowler, et al., 2020; Griffiths, Gilmore, et al., 2020; Tobler et al., 2009). The red brocket deer is a large‐bodied (12–65 kg) ruminant (Bodmer, 1989; Branan & Marchinton, 1985; Emmons & Feer, 1997; Jones et al., 2018; Robinson & Redford, 1986) that is found throughout tropical South America (Gallina‐Tessaro et al., 2019). The red brocket deer is primarily nocturnal, but often has a flexible activity pattern (Di Bitetti et al., 2008; Griffiths, Bowler, et al., 2020; Griffiths, Gilmore, et al., 2020), and is typically solitary (Reyna‐Hurtado & Chapman, 2019). It is most commonly found in *terra firme* forests, but can also inhabit swampy areas (Bodmer, 1991b; Tobler et al., 2009). The red brocket deer is primarily a frugivore (Danell et al., 2006; Lall et al., 2018; Prado, 2013) and seed predator (Gayot et al., 2004), though some small percentage of seeds may be dispersed (Bodmer, 1991b). The red brocket deer may consume browse seasonally as fruit becomes scarce (Emmons & Feer, 1997; Duarte et al., 2010). Reproduction of the red brocket deer is poorly understood, but thought to occur year‐round with about 0.76–0.82 young born per adult female per year (Gallina‐Tessaro et al., 2019; Mayor et al., 2011). The drivers of geophagy of the red brocket deer remain unknown, though its frugivorous diet suggests it may be deficient in some minerals, and its frequent visitation at mineral licks suggest it may be an important species for transporting minerals across the landscape (Doughty et al., 2016). Studies on geophagy of other cervids worldwide have attributed the behavior to a demand for mineral supplementation (Klaus & Schmidg, 1998; Lavelle et al., 2014; Weeks, 1978).

This study uses soil characteristic and camera trap data from a large number of mineral licks of Amazonian interior forest and a robust modeling approach to assess the drivers of geophagy for the red brocket deer. In particular, we test three different hypotheses posed for geophagy in the literature:

1. The demand for micro and macronutrients: the mineral supplementation hypothesis
2. The alleviation of gastrointestinal issues by alkaloid‐binding clays: the toxin adsorption hypothesis
3. The selection of mineral licks based on habitat: the habitat partitioning hypothesis

We estimated evidence for the validity of each of these hypotheses using the significance of specific soil‐ and landscape‐based covariates included in a generalized linear modeling framework.

## 2 MATERIALS AND METHODS

### 2.1 Study site

**FIGURE 1:**
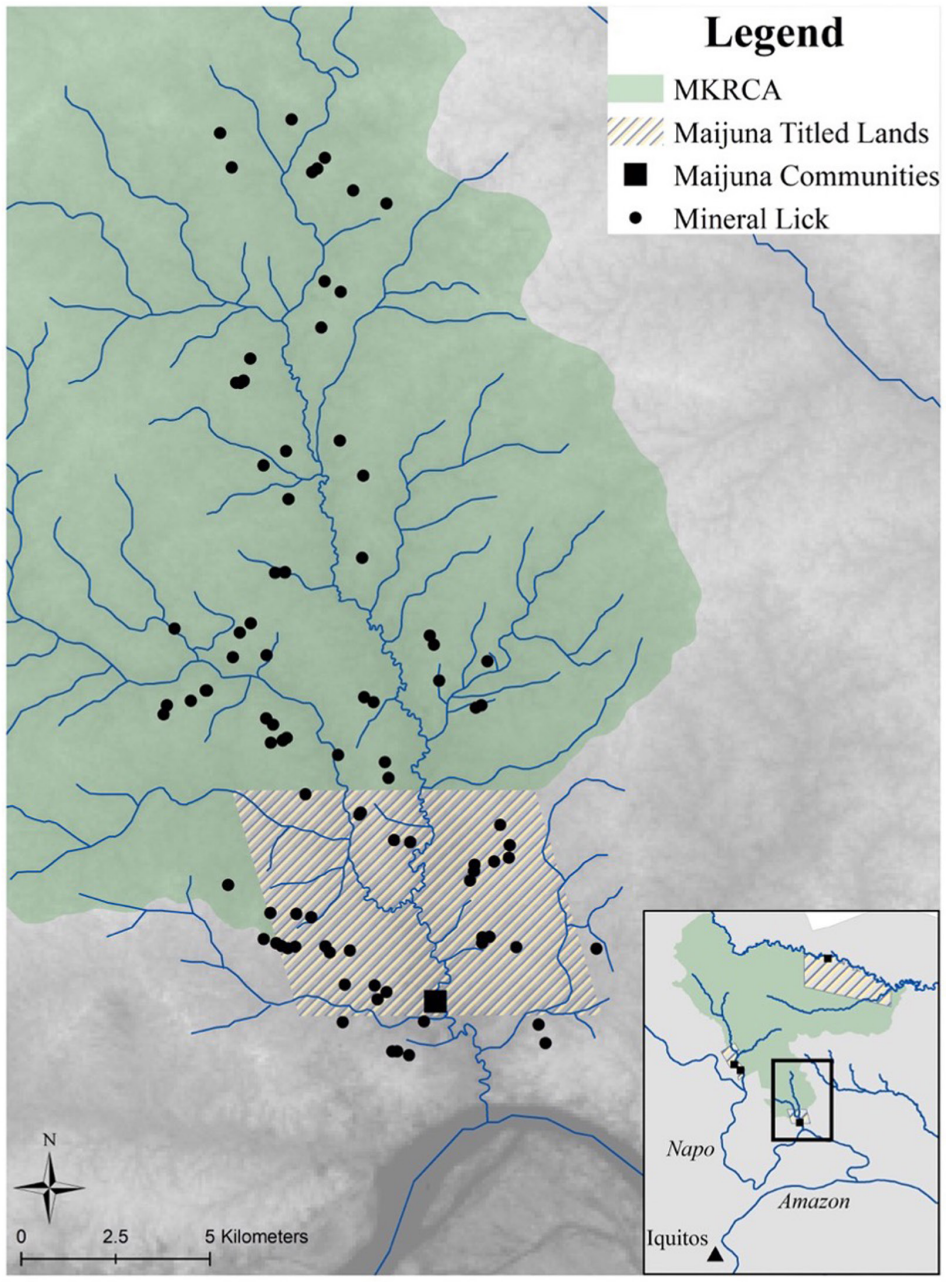
Map of the study site, the Maijuna‐Kichwa Regional Conservation Area (MKRCA) and the titled lands of the Maijuna community of Sucusari in the Peruvian Amazon. Map adopted from Griffiths, Jin, et al. (2022).

This study occurred in the Maijuna‐Kichwa Regional Conservation Area (MKRCA) and the titled lands of the Maijuna community of Sucusari, which adjoin the MKRCA to the south, in the northeastern Peruvian Amazon. The MKRCA is a 391,039‐ha protected area in the Department of Loreto, about 120 km northeast of the city of Iquitos, the capital of Loreto (Figure 1). The titled lands of the community of Sucusari encompass 4771 ha. The dominant habitat types in the region are primary, upland *terra firme* and seasonally flooded forests. The study area was completely encompassed by the watershed of the Sucusari River, a tributary of the Napo River, which has an area of about 508 km^2^. The region is characterized by a mean annual precipitation of 3100 mm and a mean annual temperature of 26°C (Marengo, 1998). There is a marked wet season that runs from November to May, with the dry season running from June to October (Espinoza Villar et al., 2009).

### 2.2 Camera trapping at mineral licks

These camera trapping methods were previously used to assess temporal visitation patterns of mammals (Griffiths, Bowler, et al., 2020; Stewart et al., 2022), natural history insights (Griffiths, Gilmore, et al., 2020) and dissimilarities among species assemblages (Griffiths et al., 2021) at mineral licks, along with the development of allometric relationships between mammal body mass and sodium deficiency (Duvall et al., 2023).

We set camera traps (Bushnell Aggressor, Boly Scout Guard) at 80 mineral licks in the Sucusari River basin from August, 2018 to June, 2019 in a series of four deployments that each lasted 60–80 days (Kays et al., 2020) across the dry and wet seasons (Griffiths, Bowler, et al., 2020). Up to 25 mineral licks were camera trapped in a single deployment before the cameras were rotated to new mineral licks. Mineral licks chosen for deployments were initially randomly selected; for the last deployment, mineral licks with less sampling effort due to camera malfunctions were prioritized. After the sampling period, we eliminated all mineral licks from the sample that did not achieve a total sampling effort of 55 camera nights, since mineral licks with fewer camera nights did not meet the sampling criteria for species richness established by Kays et al. (2020), leaving 52 mineral licks for analysis in this study.

At each mineral lick, we set camera traps facing the active faces of the lick, which were determined by looking for signs of recent animal activity (e.g. teeth and claw marks; Tobler et al., 2009). Camera traps were set 50 cm from the ground on burst photo mode, taking a set of three rapid‐fire images each time the camera was triggered (Tobler et al., 2008). Mineral licks with multiple active faces received more than one camera trap to achieve coverage of all active areas of the lick; the mean number of camera traps per lick was 1.2. The mineral licks in this study typically had saturated soil and often had standing water, regardless of season, with the lick area itself devoid of vegetation (Griffiths, Bowler, et al., 2020; Griffiths, Gilmore, et al., 2020).

We identified all red brocket deer in camera trap images, removed empty images and organized data for analyses using CameraBase v1.7 (Tobler, 2015). The number of individuals in the same photograph was also recorded. Sets of images were binned into independent visitation events, where multiple detections of red brocket deer within 1 h of each other were considered one visitation event at the mineral lick, following Tobler et al. (2008).

### 2.3 Soil sample collection and analysis

These soil collection and analysis methods were previously used to describe fundamental physical and chemical differences between soils from mineral licks and control soils (Griffiths, Jin, et al., 2022).

We revisited all mineral licks in the study in January 2022 to collect soil samples. At each mineral lick, a Maijuna expert and a trained research assistant collected soil from the feeding site of each lick, which was identified by looking for teeth and claw marks. Approximately 1 kg of soil was collected from feeding sites by scraping horizontally across exposed soil with a clean trowel (Mahaney & Krishnamani, 2003). Soil samples were placed in airtight, labeled plastic bags. In mineral licks where there were multiple active feeding sites with visibly distinct soil characteristics, one sample was taken from each feeding site, and these samples were analyzed separately. We then collected two samples of control soils by walking 5 m in a random direction, outside of the lick area devoid of vegetation. We scraped away organic debris from the topsoil and excavated 1 kg of subsoil (Mahaney & Krishnamani, 2003). We air dried all soil samples in the shade until they were no longer saturated, then they were rebagged and labeled for analysis. Soil samples were analyzed for their physical and chemical characteristics at SGS Peru in Lima, Peru (see Griffiths, Jin, et al., 2022).

### 2.4 Data analysis

We summarized red brocket deer visitation at mineral licks by calculating summary statistics of visit frequency (visits per 100 camera nights) at all mineral licks in the sample that had camera traps and red brocket deer visited at least once.

We used a generalized linear modeling approach to assess the drivers of geophagy of the red brocket deer. First, we binned soil samples from the same mineral lick by taking the mean of all relevant soil characteristics, leaving one “consumed” sample and one control sample at each mineral lick to account for pseudoreplication. We removed “consumed” samples (samples from feeding sites) which were never camera trapped since it is not clear whether red brocket deer would have visited those sites, leaving a total sample size of *n* = 129. We assigned a visit frequency to each consumed sample, which was the number of visits by red brocket deer per 100 camera nights at that mineral lick. All control soils were assigned a visit frequency of 0. If a mineral lick was camera trapped but never visited by red brocket deer (*n* = 6 mineral licks), a visit frequency of 0 was also assigned to those consumed samples. We used visit frequency as our response variable for modeling.

We constructed a global zero‐inflated model with two components: a binomial distribution to test the likelihood of a soil being consumed, and a Poisson distribution to test the predicted visit frequency of samples that were consumed. The covariates in the model were soil characteristics that were hypothesized to have some effect on red brocket deer physiology and landscape‐level mineral lick characteristics that may affect deer movement and mineral lick selection (Table 1; Tobler et al., 2009). Elevation, surface roughness, and slope were calculated using LANDSAT Collection 2 data. We also included interactions between exchangeable Ca and free P, and concentrations of Cu and Zn, Fe and Mn, and Cu and Fe since these are known to interact in the digestive systems of cervids, each impacting the absorption of the other (National Research Council (NRC), 2007). All covariates were tested for collinearity before including them in the model, with a correlation cutoff of *r* ^2^ > .7 for inclusion (Dormann et al., 2013). All continuous covariates were scaled before including them in the model.

**TABLE 1:** Specific covariates tested in zero‐inflated models and the hypothesis for geophagy that the specific covariate relates to.

Covariate tested	Hypothesis addressed
Exchangeable Na (meq/100 g)	1. Mineral Supplementation
Exchangeable Mg (meq/100 g)	1. Mineral Supplementation
Cu (ppm)	1. Mineral Supplementation
Exchangeable K (meq/100 g)	1. Mineral Supplementation
Exchangeable H (meq/100 g)	1. Mineral Supplementation
Soluble B (ppm)	1. Mineral Supplementation
Zn (ppm)	1. Mineral Supplementation
Mn (ppm)	1. Mineral Supplementation
Free P (ppm)	1. Mineral Supplementation
Fe (ppm)	1. Mineral Supplementation
Exchangeable Al (meq/100 g)	1. Mineral Supplementation
Total N (%)	1. Mineral Supplementation
Exchangeable Ca (meq/100 g)	1. Mineral Supplementation
	2. Toxin Adsorption
Clay content (%)	2. Toxin Adsorption
CEC (meq/100 g)	2. Toxin Adsorption
pH (1:1)	2. Toxin Adsorption
Elevation (m)	3. Habitat Partitioning
Slope (degrees)	3. Habitat Partitioning
Surface roughness (m)	3. Habitat Partitioning
Distance from water (km)	3. Habitat Partitioning

We used a backwards‐stepwise model selection process (Murtaugh, 2009), dropping one covariate at a time until dropping any more covariates resulted in a higher AIC, yielding the most parsimonious optimal model. Once the optimal model was selected, we tested covariates that were correlated and included in the optimal model (e.g., pH and exchangeable Ca) to see which lowered the AIC of the optimal model the most and should be included; however, in those cases, both covariates are discussed. Model fit was tested by visually examining residuals.

All analyses were conducted in R (version 4.2.1; R Core Team, 2022). Zero‐inflated models were constructed using the *zeroinfl* function in the *pscl* package (Jackman, 2020; Zeileis et al., 2008).

## 3 RESULTS

Red brocket deer visited 46 of the 52 mineral licks that had camera traps. The mean visit frequency at these sites was 37.17 (SD = 45.80) visits per 100 camera nights, with a range of 1.12–205.10 visits per 100 camera nights. On average, red brocket deer spent 9.57 min at mineral licks during a given visit, with a minimum mean visit duration of 2 min and a maximum mean visit duration of 41.02 min at a mineral lick.

**FIGURE 2:**
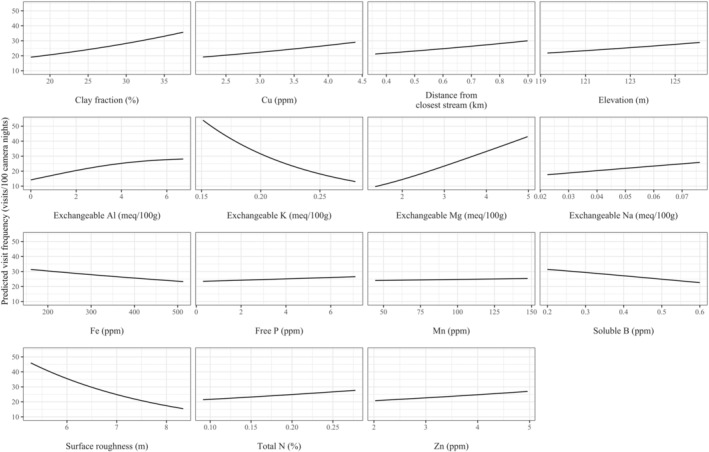
Optimal zero‐inflated model results showing the effects of mineral lick characteristics on the predicted visitation rates of the red brocket deer at Amazonian mineral licks. For display purposes, all covariates in the model that do not appear on individual graphs were held at the mean for consumed soils. Variables on *x*‐axes range across the inner quartile range of consumed soils.

The optimal model of drivers of geophagy of the red brocket deer included exchangeable Mg, Na, and Al, soluble B, and the interaction between Mg and P in the binomial half of the model. Many covariates were included in the Poisson half of the optimal model: soluble B, exchangeable Mg, K, Na, and Al, free P, total N, concentrations of Cu, Fe, Mn, and Zn, clay fraction, elevation, surface roughness, distance from the closest stream, and interactions between Mg and P, Fe and Mn, Cu and Fe, and Cu and Zn (Table 2). Soluble B, free P, exchangeable K and Al, concentration of Fe, and surface roughness all had a negative effect on predicted red brocket deer visitation while all other covariates had a positive effect. Red brocket deer visitation was predicted to increase dramatically at high concentrations of exchangeable Mg (Figure 2). Visitation was predicted to decrease to close to zero at exchangeable K concentrations greater than about 0.25 meq/100 g and soluble B concentrations greater than 1 ppm when all other covariates were held constant. Clay fraction, concentration of Cu, exchangeable Al, exchangeable Na, concentration of Mn, total N, and concentration of Zn all had a relatively moderate positive effect on predicted visitation (Figure 2). Overall, concentration of Fe, free P, and distance from the closest stream had a relatively small effect on predicted red brocket deer visitation (Figure 2). Red brocket deer were predicted to visit licks more frequently at higher elevations and lower surface roughness.

**TABLE 2:** Optimal zero‐inflated model results testing hypotheses for the drivers of geophagy in the red brocket deer at Amazonian mineral licks.

Covariate	Coeff. Est.	SE	*z*	*p*
Poisson				
**Soluble B (ppm)**	**−0.597**	**0.042**	**−14.276**	**.000**
**Exchangeable Mg (meq/100 g)**	**0.314**	**0.071**	**4.447**	**.000**
Free P (ppm)	−0.058	0.033	−1.743	.081
**Exchangeable K (meq/100 g)**	**−0.986**	**0.067**	**−14.720**	**.000**
**Exchangeable Na (meq/100 g)**	**0.143**	**0.032**	**4.533**	**.000**
**Total N (%)**	**0.217**	**0.036**	**6.038**	**.000**
Exchangeable Al (meq/100 g)	−0.089	0.053	−1.667	.096
**Cu (ppm)**	**0.407**	**0.054**	**7.516**	**.000**
**Fe (ppm)**	**−0.147**	**0.060**	**−2.468**	**.014**
Mn (ppm)	0.042	0.038	1.105	.269
**Zn (ppm)**	**0.483**	**0.051**	**9.450**	**.000**
**Clay fraction (%)**	**0.350**	**0.062**	**5.622**	**.000**
**Elevation (m)**	**0.227**	**0.044**	**5.110**	**.000**
**Surface roughness (m)**	**−0.720**	**0.056**	**−12.943**	**.000**
**Distance from closest stream (km)**	**0.209**	**0.046**	**4.583**	**.000**
**Exchangeable Mg x Free P**	**0.214**	**0.032**	**6.672**	**.000**
Cu × Fe	−0.081	0.042	−1.923	.055
**Fe** × **Mn**	**−0.190**	**0.042**	**−4.552**	**.000**
**Cu** × **Zn**	**−0.368**	**0.061**	**−6.083**	**.000**
Binomial				
**Soluble B (ppm)**	**−0.934**	**0.430**	**−2.172**	**0.030**
**Exchangeable Mg (meq/100 g)**	**−1.597**	**0.380**	**−4.204**	**0.000**
Free P (ppm)	−0.332	0.278	−1.196	0.232
**Exchangeable Na (meq/100 g)**	**−1.228**	**0.526**	**−2.335**	**0.020**
**Exchangeable Al (meq/100 g)**	**−1.513**	**0.365**	**−4.150**	**0.000**
Exchangeable Mg × Free P	0.600	0.319	1.882	0.060

*Note*: Statistically significant covariates shown in bold.

**FIGURE 3:**
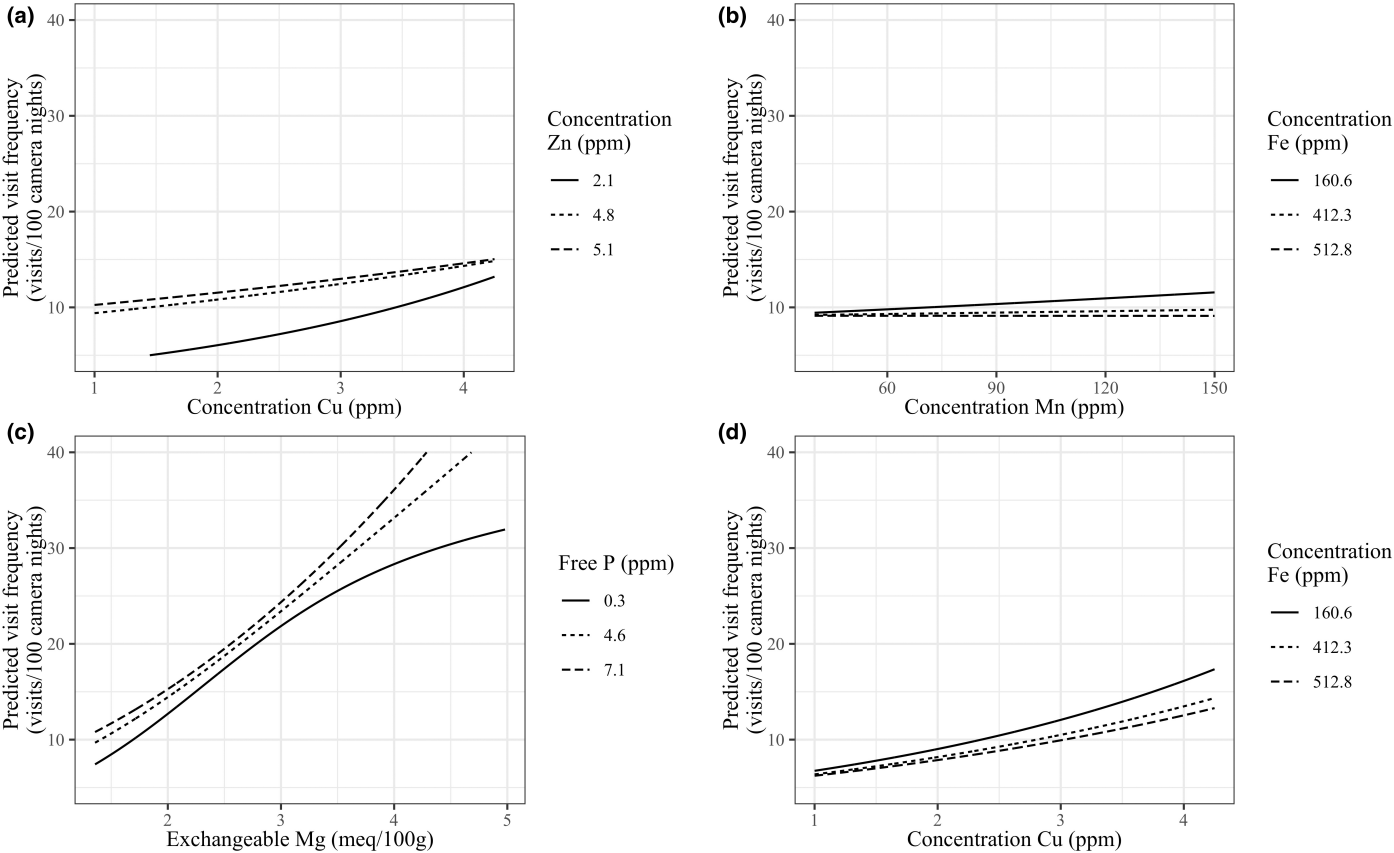
The interaction between (a) Cu and Zn, (b) Mn and Fe, (c) Mg and P, and (d) Cu and Fe concentrations on predicted red brocket deer visitation at Amazonian mineral licks. All other covariates in the model held at the mean for display purposes. Variables on *x*‐axes range across the inner quartile range of consumed soils.

Four interactions were included in the optimal model: concentrations of Cu and Zn, Mn and Fe, Cu and Fe, and exchangeable Mg and free P. Predicted visitation was highest at high Cu and low Zn concentrations. However, at very low Cu concentrations, predicted visitation was slightly higher at sites with higher Zn concentrations (Figure 3a). The influence of Mn concentration on predicted visitation was highest at low Fe concentrations. At high Fe concentrations, Mn concentration had a negative effect on predicted visitation (Figure 3b). Exchangeable Mg and free P were included as an interaction since Ca and P are known to interact (NRC, 2007) and exchangeable Ca and Mg were highly correlated. The inclusion of Mg over Ca lowered the AIC of the optimal model significantly, but the interaction here also signifies the interaction between Ca and P. Predicted visitation increased most rapidly at high concentrations of both Mg and P, and the influence of exchangeable Mg on predicted visitation was lowest at low free P concentrations (Figure 3c). The influence of Cu concentration on predicted visitation was highest at low Fe concentrations, acting similarly to Mn (Figure 3d).

Overall, two models fell within ∆AIC < 2 of the optimal model, one included Mn concentration (∆AIC = 0.128) and the other included both Mn concentration and exchangeable K (∆AIC = 1.294; Table S1).

## 4 DISCUSSION

This study is the first of its kind to use a large sample size of mineral licks in the same watershed (*n* = 52) camera trap records (*n* = 1733 independent visits) and soil samples (*n* = 218), in a robust modeling approach to test the three main hypotheses of geophagy for this cryptic cervid. Our results demonstrate the ecological significance of mineral licks for frugivorous species and provide substantial evidence for the mineral supplementation hypothesis.

The spatial variation in deer visitation, and the large variability in soil characteristics between mineral licks, emphasizes the importance of assessing a large sample size of mineral licks when drawing inferences about geophagy. In this study, we present results from a large sample size of mineral licks spread across the entirety of the Sucusari River watershed. Our soil samples likely encompass the full range of mineral lick characteristics available to red brocket deer in this watershed. The variation between licks has likely contributed to the incomplete understanding of the drivers of geophagy in the Amazon between studies with small sample sizes (Brightsmith et al., 2008; Brightsmith & Muñoz‐Najar, 2004; Emmons & Stark, 1979; Ghanem et al., 2013; Gilardi et al., 1999; Izawa, 1993; Molina et al., 2014). The limited sample sizes presented in other studies demonstrate the importance of increasing sampling to seek a larger sample size, which could be accomplished by working with local communities to identify these critical ecological sites.

In this study, we report high levels of collinearity between some soil characteristics, in particular Mg, Ca, and pH. Considering collinear variables together in the same set of analyses may cloud the interpretation of results (Dormann et al., 2013). In this case, we removed collinear covariates in our global model testing drivers of geophagy. Our optimal model showed that Mg may be the most important driver of geophagy. Since pH and Ca concentration were highly correlated with Mg, we can assume that these characteristics are also important though they were excluded due to collinearity. Testing these covariates together may have led to the mistaken assumption that the major cations Mg and Ca, along with pH were the only significant factors in driving geophagy.

Similarly, we account for any potential lack of independence in soil samples by taking the mean values within sample types for the models. In this case, because we took multiple soil samples from the same lick site (Mahaney & Krishnamani, 2003), the samples may not be independent, a condition which invalidates inferences from many approaches that have been used in other studies (e.g. basic descriptions of soils: De Souza et al., 2002; Izawa, 1993; Montenegro, 1998; correlations: Molina et al., 2014; Wilcox's rank sum test: Moe, 1993). We suggest that some of the variation in results from other studies is due to the lack of separation of collinear covariates and a lack of independence. We recommend that future studies use statistical methods that account for these complexities (e.g. PCA: Brightsmith et al., 2008; Razali et al., 2020; Sim et al., 2020).

The covariates that were predicted to cause the largest increase in visitation across their distributions were exchangeable Mg, exchangeable Na, Cu, and Zn. Mg is associated with bone growth, muscle development, and activation of enzymes in mammals (NRC, 2007), and high concentrations of Mg have been shown to increase absorption of Ca in horses (Hintz & Schryver, 1973). Mg, like Cu, has been associated with greater immune response in white‐tailed deer (Nichols et al., 2016). High concentrations of K have been shown to limit Mg absorption, a condition known as grass tetany (Ram et al., 1998). It's possible that if some fruits eaten by the red brocket deer are high in K, red brocket deer would need to supplement Mg in their diet or risk tetany (Kaspari, 2020). Accordingly, increased exchangeable K was associated with a decrease in predicted visitation. This model shows good evidence that Mg may be a major driver of mineral lick selection for the red brocket deer.

Cervids, including deer, elk, and moose, are known to have a relatively high need for Ca, Na, and P, particularly for bone and antler formation and growth and during lactation (Carciofi & Saad, 2001; Hellgren & Pitts, 1997; Landete‐Castillejos et al., 2007; Tajchman et al., 2018). While these animals may have similar physiological needs to red brocket deer, each animal's needs are determined by deficiencies in their diet, and the red brocket deer's frugivorous diet varies from the grazing/browsing diets of most other cervids. In the Amazon, a frugivorous diet is linked with lower micronutrient content than a grazing or browsing diet (Doughty et al., 2016; Ghanem et al., 2013). In general, supplementation of major cations can also improve rumen buffering (Beede & Collier, 1986). Cervids, in general, are particularly vulnerable to hypocalcaemia and hypophosphataemia while growing. While the red brocket deer has relatively small antlers compared to other cervid species, its highly frugivorous diet (Bodmer, 1989; Duarte et al., 2010) suggests that it may be deficient in some or all of the macro and micro minerals needed for growth. While both Ca and P are needed for growth, high concentrations of P can limit Ca absorption since P and Ca both compete for the same binding sites (NRC, 2007). Of particular importance, then, is the Ca:P ratio, and a Ca:P ratio of about 12:58–59 (or 0.2) has been considered optimal for cervids (Perkins, 1991). Interestingly, many of the consumed soils in this study were at or close to the optimal Ca:P ratio of 0.2, but many unconsumed and control soils were also near, or at, the optimal ratio. The interaction between Mg (and therefore Ca as well due to high correlation) and P was significant, indicating that the Ca:P ratio may be a determinant in mineral lick selection.

Na is a key mineral for herbivore growth, survival, and reproduction but is present in very low quantities in vegetation and fruit in the Amazon (Doughty et al., 2016). Many studies have described an association between Na content and mineral lick visitation (Brightsmith & Muñoz‐Najar, 2004; De Souza et al., 2002; Dudley et al., 2012; Lavelle et al., 2014; Stephenson et al., 2011), and Na deficiency has been a leading hypothesis for geophagy for decades. Our models describe a positive association between exchangeable Na and predicted visitation for the red brocket deer, adding to this body of literature. Kaspari (2020) describes how Na may also facilitate P uptake in ruminants which have P‐demanding gut microbiomes. Even compared to other native herbivorous and frugivorous species, including other ungulates, the red brocket deer's diet and body size suggest it may be the most deficient in Na (Duvall et al., 2023).

Our models predicted that mineral licks that had a Cu concentration of more than 10 ppm were almost guaranteed to be consumed by red brocket deer, and that proportion increased significantly when concentrations of Fe were relatively low. High levels of Fe have been shown to decrease absorption of Cu (NRC, 2007). Our models demonstrated this interaction between Cu and Fe, where the probability of soil consumption was the highest when soils were simultaneously rich in Cu and relatively poor in Fe. These results indicate that red brocket deer may select geophagical soils based on Cu content and the ratio of Cu:Fe and/or Cu:Zn to maximize Cu absorption. In cervids, Cu is most commonly associated with function of the immune system. In white‐tailed deer (*Odocoileus virginianus*), higher Cu intake was associated with less incidence of chronic wasting disease (Nichols et al., 2016) and a higher immune response to pathogens (Bartoskewitz et al., 2007). In red deer (*Cervus elaphus*), low levels of Cu were associated with lower carcass weight (Handeland et al., 2017). While other studies have measured Cu concentrations in lick soils (Emmons & Stark, 1979; Ghanem et al., 2013; Gilardi et al., 1999; Molina et al., 2014; Montenegro, 2004; Powell et al., 2009), our study is the first study to propose that Cu, and the relationship between Cu and Fe and Cu and Zn, may be an important driver of geophagy for red brocket deer.

Concentrations of exchangeable Al were significant in the optimal model, with relatively high concentrations of Al associated with a plateau in predicted visitation. In one study in North America, cervids were observed to avoid mineral licks with high levels of Al, potentially to avoid toxicity (Lavelle et al., 2014). Given that Al can be toxic to mammals, and is loosely negatively correlated with pH, we would expect high levels of Al to be negatively associated with consumption probability. The majority of consumed samples had an Al concentration of 0. The positive relationship and significance we show here is likely an artifact of the data, where some consumed samples had very high Al concentrations, skewing the results. Since some consumed soils also had very high levels of Na, P, Cu, and pH, the importance of these soil characteristics may also be overstated by models, but also match our hypotheses. It is also possible that elements co‐exist in the soil and animals are not able to find soils that contain only the minerals that they seek; the consumption of high Al content soils may be a consequence of seeking a mineral that co‐occurs with Al. We found that only Mg, Ca, and pH were highly correlated, but it is possible that this relationship between minerals occurs in specific mineral licks. However, the importance of Al in geophagous soils has previously been stated for primates (Ferrari et al., 2008), so it is possible that the high Al content is a legitimate driver of geophagy in other herbivores as well.

Overall, high intakes of many minerals, including Ca, P, Mn, and Cu can be toxic to cervids (Lavelle et al., 2014; McDowell, 2003). Similarly, high levels of several minerals can influence absorption of other minerals (NRC, 2007). In this study, we tested several of those interactions: Mg (and Ca) and P, Cu and Fe, Fe and Mn, and Cu and Zn. All of these interactions appeared in the optimal model and are known to interact in the digestive systems of ruminants (Tajchman et al., 2018) which indicates that simple comparisons between consumed and control soils are too simplistic to draw conclusions from, and more complex statistical methods are needed. The influence of potential toxicity could be tested with a large enough sample size by including polynomial terms in models of geophagy. Here, we faced a lack of convergence when polynomial terms were included, indicating that a greater sample size of brocket deer mineral licks are needed to test toxicity. However, it may be that brocket deer avoid ingesting toxic amounts of minerals simply by consuming a smaller quantity of soil.

Soil pH, correlated with Mg, also has a significant association with geophagy. This result is in line with other studies, which have posited that a main driver of geophagy is the detoxification of dietary alkaloids that were consumed (Freeland & Janzen, 1974; Gilardi et al., 1999; Krishnamani & Mahaney, 2000; Milton, 1998). However, this hypothesis is also linked to clay content of the soil, where a higher clay content has a higher adsorption capacity due to larger surface area. In our models, clay content in the soil was associated with increased visitation, as predicted under the toxin adsorption hypothesis. This result lends evidence to the conclusion that red brocket deer may receive dual benefits from soil ingestion. It should be noted that certain species of clays, such as kaolinite, can be critical for detoxification. While kaolinite is the dominant clay type in the Iquitos region (Irion, 1984; Kauffman et al., 1998), we did not measure the structure of clays present in this study. Other studies have determined that soils may alleviate gastrointestinal issues in other species and that clay content and pH are potential drivers of geophagy (Gilardi et al., 1999; Mahaney et al., 1996) or that species may derive dual benefits (Ayotte et al., 2006; Brightsmith et al., 2008; Diamond et al., 1999; Ferrari et al., 2008; Ghanem et al., 2013; Klaus et al., 1998; Kreulen, 1985; Krishnamani & Mahaney, 2000; Molina et al., 2014; Voigt et al., 2008).

There is some evidence that animals can detect Ca and Mg in soils (McCaughey & Tordoff, 2002; Tordoff, 2001), so it may be that cervids select soils with high Ca and Mg and derive other benefits, such as Cu supplementation, from that selection. The highly frugivorous diet of the red brocket deer (Bodmer, 1989; Danell et al., 2006; Gayot et al., 2004; Prado, 2013; Duarte et al., 2010) also supports that the main driver of geophagy is likely mineral supplementation, since plant‐based alkaloids are typically found in the mature leaves of plants (Julliot & Sabatier, 1993). The red brocket deer would only be consuming minor amounts of alkaloids.

The significance of landscape features in the model demonstrates that habitat selection may be an important factor in geophagy of the red brocket deer, a result that is not in line with results from Tobler et al. (2009), who reported a lack of habitat selection at mineral licks. Bodmer (1991a, 1991b) states that red brocket deer have a preference for inclined terrain in *terra firme* forests, and this may be the cause of the significance of landscape features here.

Several other studies, from the Amazon (Brightsmith et al., 2017; Montenegro, 2004; Setz et al., 1999), North America (Jones & Hanson, 1985), and other areas of the tropics (Matsubayashi et al., 2007; Mills & Milewski, 2007; Stephenson et al., 2011) have determined that mineral supplementation was likely the driver of geophagy at mineral licks, and there has been a particular emphasis on Na (Dudley et al., 2012; Holdø et al., 2002; Lavelle et al., 2014; Moe, 1993; Powell et al., 2009; Weeks, 1978). This study uses a robust statistical analysis on a large sample of interior forest Amazonian mineral licks, and it shows strong support for the mineral supplementation hypothesis in cervids, with some evidence that deer receive dual benefits.

### CONCLUSIONS

Mineral licks are critical to the feeding ecology of Amazonian frugivores, in particular the red brocket deer. We suggest that red brocket deer receive dual benefits from mineral licks, satisfying both the mineral supplementation and toxin adsorption hypotheses, and that some selection of mineral licks based on habitat occurs. While we found evidence of all three hypotheses influencing red brocket deer visitation, our models suggest that the strongest driver of geophagy in the red brocket deer is mineral supplementation of Mg, Ca, Na, Cu, and Zn.

## AUTHOR CONTRIBUTIONS

**Brian M. Griffiths:** Conceptualization (lead); data curation (lead); formal analysis (lead); funding acquisition (lead); investigation (lead); methodology (lead); project administration (lead); validation (lead); visualization (lead); writing – original draft (lead); writing – review and editing (lead). **Lesa G. Griffiths:** Funding acquisition (equal); project administration (equal); resources (lead); writing – review and editing (equal). **Yan Jin:** Conceptualization (supporting); formal analysis (supporting); investigation (supporting); methodology (supporting); resources (supporting); writing – review and editing (equal). **Michael P. Gilmore:** Conceptualization (supporting); funding acquisition (supporting); investigation (supporting); methodology (supporting); project administration (supporting); supervision (equal); writing – review and editing (lead).

## FUNDING INFORMATION

This work was supported by a Fulbright U.S. Student Grant awarded to Brian M. Griffiths.

## CONFLICT OF INTEREST STATEMENT

The authors have no relevant financial or nonfinancial interests to disclose.

## Supporting information


Table S1.

## Data Availability

Analyses reported in this article can be reproduced using the data provided by Griffiths, Griffiths, et al. (2022).
