# Supplementary material for: Drivers of geophagy by red brocket deer (Mazama americana) at Amazonian interior forest mineral licks
Source: Ecol Evol. 2024 Feb 9;14(2):e10968. doi: 10.1002/ece3.10968 (PMC10857927; doi:10.1002/ece3.10968)
Supplement: Supplementary file 1 — Table S1. [file ECE3-14-e10968-s001.docx]

Drivers of geophagy in red brocket deer (*Mazama americana*) at Amazonian interior forest mineral licks

Supplementary Information

Figures and Tables

Table S1. Model selection results for zero-inflated generalized linear model assessing the drivers of geophagy at Amazonian interior forest mineral licks. The optimal model and competitive alternate models within ∆AIC < 2 of the optimal model are shown. Weight indicates Akaike weight.

| **Model** | **K** | **∆AIC** | **Weight** |
| --- | --- | --- | --- |
| Poisson: B + Mg + P + K + Na + N + Al + Cu + Fe + Mn + Zn + Clay + Elevation + Surface roughness + Distance from water + Mg:P + Cu:Fe + Fe:Mn + Cu:Zn  Binomial: B + Mg + P + Na + Al + Mg:P | 27 | - | 0.36 |
| Poisson: B + Mg + P + K + Na + N + Al + Cu + Fe + Mn + Zn + Clay + Elevation + Surface roughness + Distance from water + Mg:P + Cu:Fe + Fe:Mn + Cu:Zn  Binomial: B + Mg + P + Na + Al + Mn + Mg:P | 28 | 0.13 | 0.33 |
| Poisson: B + Mg + P + K + Na + N + Al + Cu + Fe + Mn + Zn + Clay + Elevation + Surface roughness + Distance from water + Mg:P + Cu:Fe + Fe:Mn + Cu:Zn  Binomial: B + Mg + P + K + Na + Al + Mn + Mg:P | 29 | 1.29 | 0.19 |
